# Mechanism Exploration of Arylpiperazine Derivatives Targeting the 5-HT_2A_ Receptor by In Silico Methods

**DOI:** 10.3390/molecules22071064

**Published:** 2017-06-26

**Authors:** Feng Lin, Feng Li, Chao Wang, Jinghui Wang, Yinfeng Yang, Ling Yang, Yan Li

**Affiliations:** 1 Key Laboratory of Xinjiang Endemic Phytomedicine Resources, Pharmacy School, Shihezi University, Shihezi 832002, Xinjiang, China; fenglin_dut@yeah.net; 2 Key Laboratory of Industrial Ecology and Environmental Engineering (MOE), Faculty of Chemical, Environmental and Biological Science and Technology, Dalian University of Technology, Dalian 116024, Liaoning, China; chaowang_dlou@163.com (C.W.); jhwang_dlut@163.com (J.W.); yinfengyang@yeah.net (Y.Y.); 3 Department of Civil Engineering, Henan Institute of Engineering, Zhengzhou 451191, Henan, China; fengli@haue.edu.cn; 4 Institute of Interdisciplinary Integrative Medicine Research, Shanghai University of Traditional Chinese Medicine, Shanghai 201203, China; yling@dicp.ac.cn

**Keywords:** depression, 5-HT_2A_ receptor, arylpiperazine derivative, 3D-QSAR, molecular docking, molecular dynamics

## Abstract

As a G-protein coupled receptor, the 5-hydroxytryptamine 2A (5-HT_2A_) receptor is known for its critical role in the cognitive, behavioural and physiological functions, and thus is a primary molecular target to treat psychiatric diseases, including especially depression. With purpose to explore the structural traits affecting the inhibitory activity, currently a dataset of 109 arylpiperazine derivatives as promising 5-HT_2A_ antagonists was built, based on which the ligand-based three-dimensional quantitative structure-activity relationship (3D-QSAR) study by using both comparative molecular field analysis (CoMFA) and comparative molecular similarity indices analysis (CoMSIA) approaches was carried out. The resultant optimal CoMSIA model displays proper validity and predictability with cross-validated correlation coefficient *Q*^2^ = 0.587, non-cross-validated correlation coefficient *R*^2^_ncv_ = 0.900 and predicted correlation coefficient for the test set of compounds *R*^2^_pre_ = 0.897, respectively. Besides, molecular docking was also conducted to investigate the binding mode between these ligands and the active site of the 5-HT_2A_ receptor. Meanwhile, as a docking supplementary tool to study the antagonists’ conformation in the binding cavity, molecular dynamics (MD) simulation was also performed, providing further elucidation about the changes in the ligand-receptor complex. Lastly, some new molecules were also newly-designed based on the above results that are potential arylpiperazine antagonists of 5-HT_2A_ receptor. We hope that the present models and derived information may be of help for facilitating the optimization and design of novel potent antagonists as antidepressant drugs as well as exploring the interaction mechanism of 5-HT_2A_ antagonists.

## 1. Introduction

Cognitive inflexibility, the inability spontaneously to sustain, withhold, or modify adaptive behavior in response to varying situational demands, is correlated with diverse psychiatric disorders, including especially depression, schizophrenia and obsessive-compulsive disorders [1]. As a matter of fact, cognitive dysfunction seems to be an independent and core domain of depression which may lead to low functioning level and reduced life quality of depression patients [2]. The disease it leads to, i.e., depression (notably major depression), threats the health of approximately 121 million people and thus is among the top five primary causes of disease and disability burden all over the world [3,4]. In fact, across the lifespan, the onset and process of depression are usually influenced by stressful life events that include loss, threat, defeat, or humiliation [4]. Some 2–5% of the US population suffer from severe forms of depression, and milder forms of the disease affect up to 20% of the population [5]. Besides, cognitive dysfunction is also recurrent, life threatening (because of the risk for suicide), and a leading cause of morbidity throughout the world [5]. Therefore, pharmacologic drugs are becoming widely desirable for treating psychiatric disorders.

Over the past few decades, the synaptic actions of monoamine neurotransmitters including serotonin (SER, 5-HT) and norepinephrine (NE) were recognized as crucial evidences to psychiatric disease [3]. 5-HT, binding to a G-protein coupled receptor (GPCR), takes part in various aspects of physiology, behavior and cognition [6]. More importantly, it is also involved in the pathogenesis of diverse diseases ranging from psychiatric disorders like depression to neurological disorders such as Alzheimer’s disease (AD) [6]. Actually, 5-HT receptors are widely considered as primary targets for cognitive enhancement in psychiatric disease [7]. Indeed, the outstanding efficacy of present atypical antipsychotics on aspects of attention including vigilance and to a degree executive functioning in psychiatric disease patients may be partly due to their indirect or direct effects on 5-HT_2A_ receptors [7]. Factually, in the treatment of negative symptoms, the blockade of 5-HT_2A_ receptors or the preferential blockade of particular subtypes of dopamine receptors was always assumed to be a relative mechanism for the efficiency of atypical antipsychotics [8]. And, it is also suggested that the antagonism of 5-HT_2A_ receptor is important for the clinical result and profile of the prototype of atypical antipsychotic agent [9]. Besides, evidence also supports the hypothesis that 5-HT_2A_ signaling is a significant factor in cognitive function [10]. Consequently, the 5-HT_2A_ receptor has already become the focus of extensive studies as a target to treat psychotic disorders [11].

Recently, the development of the serotonin antagonist/reuptake inhibitors (SARIs) has become a direction in treating depressive disorders due to the effective modulation of SARIs on the 5-HT level. Clinically, several SARI drugs for the 5-HT_2A_ have been applied, such as ritanserin, YM992, M100907, LY367265 and nefazodone. Among them, ritanserin, as the first 5-HT_2A_ receptor antagonist, has been found to have high antipsychotic activity [8,12]. Actually, it not only improves the negative symptoms when added to neuroleptics in hospitalized patients with primarily negative symptoms, but also reduces the extrapyramidal side effects when added to routine antipsychotics [8]. Moreover, some studies also found that ritanserin also disrupts potential inhibition via preexposure [8]. As to YM992, it is a novel selective 5-HT reuptake antagonist that possesses 5-HT_2A_-inhibitory properties and enhances 5-HT neurotransmission after long-term administration, which may contribute to outstanding antipanic and antidepressant activities by directly blocking the 5-HT reuptake and 5-HT_2A_ receptors [13]. With regard to M100907, it is a highly selective 5-HT_2A_ receptor antagonist with superior affinity for 5-HT_2A_ receptor, eliciting a positive response in a lot of preclinical paradigms designed to test antipsychotic activity [11]. As to LY367265, it shows higher affinity for 5-HT_2A_ receptor and 5-HT transporter than the clinically potent antidepressant, nefazodone [14]. Furthermore, the advantages of nefazodone include not only reducing the possibility of sexual dysfunction or sleep disturbance, but also treating those patients who didn’t respond to other antidepressants [15].

Despite of the usefulness on antipsychotics, these agents however still have certain side effects, including the constipation, anxiety, nausea, hopelessness and even liver failure [3,14]. And some problems about long onset time and poisoning symptom are also waiting for solution. Therefore, the exploration of more potent and safer drugs is still a necessity. Presently, a series of SARIs synthesized through molecular structure modification based on above drugs exhibited a selective and effective activity against 5-HT_2A_, with an IC_50_ value as low as 5.17 nM for the most potent compound [3]. In light of these compounds, it appears of interest to apply the ligand-based drug design techniques to explore the structural factors of these 5-HT_2A_ antagonists, which may be of help for discovery of novel efficacious anti-5-HT_2A_ drugs.

With the rapid development of computer technology, chemical biology, and molecular biology, computer simulation technology plays an prominent role in the growth of new agents [16,17]. As known to all, computer-aided drug design (CADD) can greatly raise the efficiency of developing and designing novel drugs, and thus has been used more and more diffusely in present pharmaceutical industry [18,19]. In fact, CADD approaches, like especially the 3D-QSAR, molecular docking, molecular dynamics (MD), homology modeling and pharmacophore mapping techniques, have been vastly conducted in the optimization and development of inhibitors [20,21], in which 3D-QSAR modeling has proven its efficiency in exploring the pharmacological properties of the studied molecules in modern drug discovery [22,23]. Especially, 3D-QSAR methods including comparative molecular field analyses (CoMFA) [24] and comparative molecular similarity indices analyses (CoMSIA) [25] have been successfully utilized to obtain insights into the structural requirements that affect their biological activity for many series of molecules, such as heteroarylnitrile, 1,7-diazacarbazole and peptidomimetic derivatives as falcipain-2, checkpoint kinase 1 and 3C-like protease inhibitors, respectively [16,17,26]. In this study, we employed a sets of CADD approaches, including CoMFA and CoMSIA analyses, molecular docking and MD simulation with purpose to (1) probe the crucial structural factors of arylpiperazine scaffold-based potent 5-HT_2A_ antagonists [3]; (2) understand the probable binding modes at the amino acid residue level; (3) further elucidate the changes in the ligand-receptor complex embedded into the lipid bilayer; (4) design several new potential arylpiperazine antagonists of 5-HT_2A_ receptor based on the above results. The obtained findings can not only guide rational structural modification and design of novel and more potent 5-HT_2A_ antagonists, but also offer some reference for experiment study.

## 2. Materials and Methods

### 2.1. Data Set and Biological Activities

After eliminating those compounds with unspecified antagonistic activity, a total of 109 arylpiperazine derivatives targeting 5-HT_2A_ receptor were used as a dataset with their biological activities (IC_50_ values) taken from the same source [3]. For improving the normal distribution of the experimental data points, the activities of all the molecules were converted into consistent pIC_50_ (−logIC_50_) values (ranging from 5.995 to 8.287), which were used as the dependent variables in the QSAR regression analysis. In an approximate ratio of 3:1, the compounds were separated into a training (82 compounds) set to construct 3D-QSAR models and a test (27 compounds) set to validate the models. To ensure that the predictive power of the models be effectively evaluated, the selection of the test set molecules follows the rule that their pIC_50_ values are randomly but uniformly distributed in the range of the values of the entire set. All information of the 109 molecules used in this work is provided in supporting information Table S1, where sixteen representative molecules with structures and IC_50_ values are shown in Table 1.

### 2.2. Molecular Modeling and Alignment

All 3D-QSAR and molecular studies were conducted by Sybyl 6.9 package (Tripos Associates, St. Louis, MO, USA). Partial atomic charges were calculated by using the Gasteiger-Hückel method [27], and then the conformer of each compound was energy-minimized using the Tripos molecular mechanics force field [28] and the Powell conjugate gradient minimization algorithm with the convergence criterion set to 0.05 kcal·mol^−1^·Å^−1^ to ensure the stability of the conformation.

**Figure 1:**
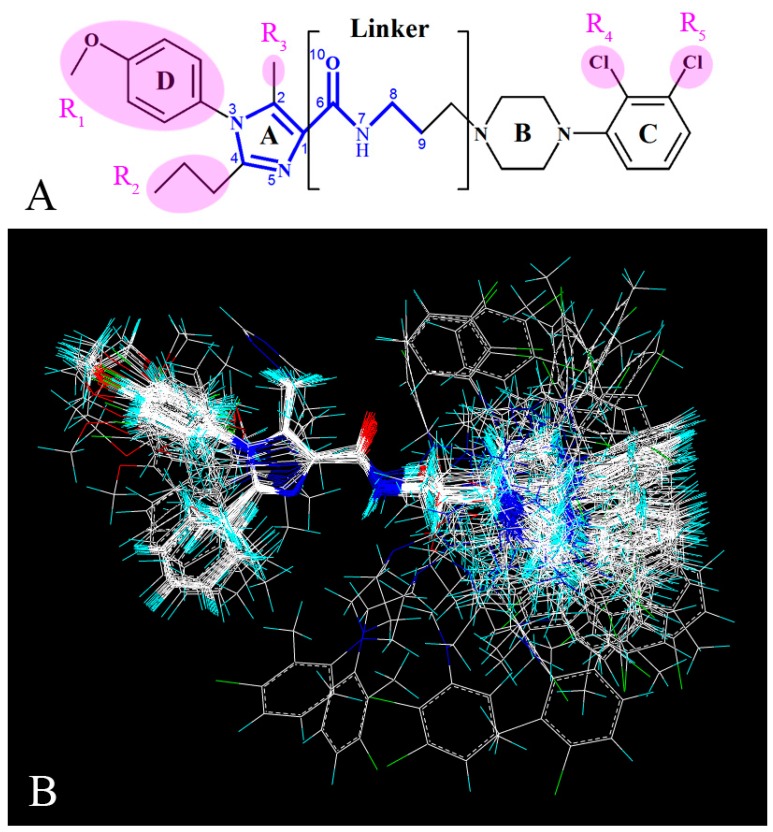
Molecular alignments of all compounds in the data set. (**A**) The common structure of molecules based on template compound **13** is displayed in bold; (**B**) The resultant alignment model.

Since in 3D-QSAR studies, the most critical step is the molecular alignment [29], presently all 3D-QSAR statistical models were constructed based on the alignment of all the molecules, and compound **13** was chosen as the template due to its most potent activity in the data set. All the molecules were fitted into the template using the “Align Database” command in Sybyl. The common skeleton in the molecular superimposition is displayed in bold in Figure 1A,B depicts the resultant model.

### 2.3. CoMFA and CoMSIA Studies

To analyze the quantitative relationship between 3D structural features and the biological activity for a set of molecules, CoMFA and CoMSIA analyses were utilized for these antagonists after conformational alignment. All superimposed molecules were placed in a 3D lattice with spacing of 2.0 Å. CoMFA fields including the steric and electrostatic fields were generated by using sp^3^ C-atom probe with a formal charge of +1.0 at each lattice point and a van der Waals (vdW) radius of 1.52 Å [30]. And both the steric and electrostatic fields were calculated by CoMFA standard method with energy cut-off values of 30.0 kcal·mol^−1^ [31]. CoMSIA is, though, an extension of CoMFA, it also includes extra hydrophobic, hydrogen bond (H-bond) donor and H-bond acceptor descriptors besides the steric and electrostatic descriptors. CoMSIA similarity index descriptors were derived by the same lattice boxes as those used in CoMFA calculations. And five different similarity descriptors were calculated by using a probe atom of charge +1.0, radius 1.0 Å. A Gaussian function was used to evaluate the mutual distance between each molecule atom and the probe atom, with no cut-off limits in CoMSIA study.

In order to obtain statistically significant 3D-QSAR models and to analyze the relationship between their biological activities and the variations in CoMFA-CoMSIA interaction energies, partial least-squares (PLS) regression analyses were conducted [32,33]. PLS can reduce an originally large number of descriptors to some principal components which are linear combinations of the initial descriptors [34]. In the present study, the CoMFA-CoMSIA descriptors were used as independent variables, while dependent variables were the pIC_50_ values. In PLS analysis, the leave one out (LOO) method that one molecule is removed from the data set and its activity is predicted by a model derived from the remainder of the data set, was used to evaluate the reliability of model by calculating the conventional correlation coefficient (*Q*^2^), the standard predicted errors (SEP) and the optimum number of components (ONC). The ONC was applied in the final non-cross-validation analysis, and the Pearson coefficient (*R*^2^_ncv_), standard error of estimate (SEE) and *F* value were calculated [34]. *Q*^2^ and *R*^2^_ncv_ as statistical index of the model’s predictive power provide useful internal metrics based on the training set. To evaluate the COMSIA models’ predictive power, an independent test set was used. In the above study process, CoMSIA similarity indices, *Q*^2^ and the predicted *R*^2^ (*R*^2^_pre_) values were calculated according to formulas presented in our previous works [21,35,36]. Finally, all 3D-QSAR results were graphically shown by field contour maps with the field type “Stdev*Coeff”.

### 2.4. Molecular Docking

Molecular docking is the method used to explore the interaction between the receptor and its ligands. It may efficiently predict the potential ligand binding sites of the protein [37]. Presently, molecular docking analysis was performed with Gold (Genetic Optimization for Ligand Docking) 5.1 [38]. Due to that an accurate 3D structure of the receptor is essential for docking analysis, and until now an X-ray structure of 5-HT_2A_ receptor is, yet, still unavailable, the homology modeling becomes a necessity in the present work. In addition, since, to our best knowledge, almost all (which is actually, 7 out of 9 as shown in Table 2) current crystal structures of 5-HT_2A_ receptor built by homology modeling for further docking analysis were based on the structure of β_2_-adrenergic receptor as a template [39,40,41,42,43,44,45,46], for purpose of a well comparison with previously-reported docking studies of known 5-HT_2A_ antagonists, the structure of 5-HT_2A_ receptor we used presently still adopted the structure of β_2_-adrenergic receptor as the homology modeling’s template. Actually, this structure was built by Ísberg et al., where the 5-HT_2A_ receptor model was constructed using the β_2_-adrenergic receptor (PDB entry 2RH1) as the main template, and then further modified to incorporate template features from the active G-protein-bound opsin crystal structure (PDB entry 3DQB) [47]. Using the protein structure as the template, hydrogen was added and the exogenous ligand was removed from the system. The probable binding conformation was then determined using the Gold suit. In fact, after a predocking process, the validated binding cavity was created by an automatic mode with specified atom coordinates. Thereafter, all compounds were docked into the binding cavity and 10 possible active docking conformations for each compound were obtained with different scores.

### 2.5. Molecular Dynamics

In order to obtain accurate model and to verify the docking result, MD simulation was carried out applying the GROMACS software package [48]. Since 5-HT_2A_ receptor is a member of GPCR possessing seven transmembrane helices, the ligand-receptor complex was embedded into an explicit dioleoylphosphatidylcholine (DOPC) lipid bilayer to relax the system [49,50]. The lipid bilayer system was generated by the Membrane Builder tool in CHARMM-GUI [23,51]. Similar to experimental conditions, the system was solvated applying TIP3P water model and NaCl ions were added to reach zero charge [52]. The simulated system was firstly subjected to energy minimization without constraints using the steepest-descent algorithm and then the system was equilibrated at 300 K via 500 ps MD simulations. Finally, a 50,000 ps simulation was carried out to maintain the stability of the whole system with a time step of 2 fs. In this process, electrostatic interactions were calculated by the particle mesh Ewald (PME) method [53], and covalent bonds involving H-atoms were constrained by the linear constraint solver (LINCS) algorithm [54]. The cut-off distances for calculating vdW and Coulomb interactions were 1.4 and 1.0 nm, respectively. Both energy minimization and MD simulation were performed under periodic boundary conditions with temperature ensemble at 300 K and normal pressure. The temperature was kept constant using the Berendsen thermostat, and the isothermal compressibility value was set to 4.5 × 10^−5^ bar^−1^ while the pressure was maintained at 1.0 bar by the Parrinello-Rahman scheme [55].

## 3. Results

### 3.1. 3D-QSAR Statistical Analysis

**Figure 2:**
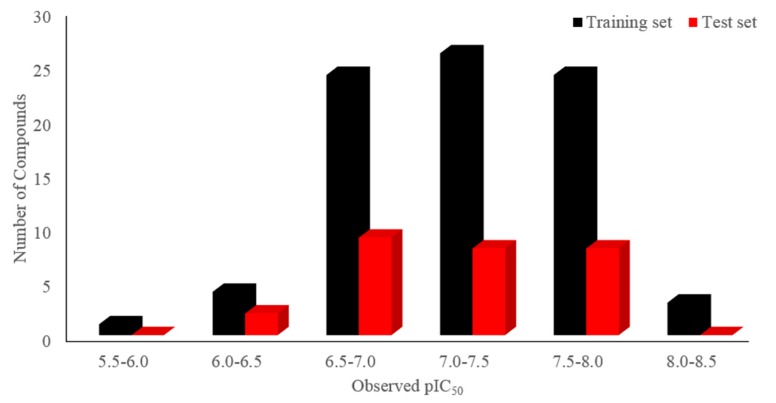
Distribution of the number of compounds versus their activities (pIC_50_) of the training (colored as black) and the test (red) sets.

Molecular alignment is considered to be a prominent factor impacting the quality of the 3D-QSAR model [17], and the three-dimensional shape of the ligand influences greatly on its interaction with the acceptor, so the ligand-based alignment was applied here to superimpose all of the 109 compounds. The entire dataset was randomly separated into a training (82 compounds) set and a test (27 compounds) set. Figure 2 displays the distribution of the number of compounds versus their activities (pIC_50_) of the dataset, in which the training and the test sets are respectively colored as black and red. Then, the overlapped compounds in the same training set were employed for both CoMFA and CoMSIA analyses. For purpose of measuring the predictive ability of a model, PLS analysis and LOO cross-validation method were used. During the modeling process, all statistical parameters were analyzed to evaluate the reliability of the models, including the LOO cross-validated *Q*^2^, non-cross-validated *R*^2^_ncv_, *R*^2^_pre_ for the test set of compounds, *F*-statistics, SEE, SEP and ONC. The obtained results of both CoMFA and CoMSIA models were listed in Table 3.

Generally, a *Q*^2^ value above 0.5 is regarded as a sign of admissible internal predictive ability. Besides, the high *R*^2^_ncv_ and *F* but low SEE values are also expected for a reliable QSAR model [19]. In the present work, by means of PLS statistical analysis, the resultant CoMFA model obtained by using both the steric and electrostatic field descriptors is unsatisfied with *Q*^2^ value of 0.496, revealing a relatively poor internal predictability. On the other hand, five field descriptors of CoMSIA (steric, electrostatic, hydrophobic, H-bond donor and H-bond acceptor) and their every possible composition were applied to build models, respectively. Among the derived 31 models, the CoMSIA model built by the combination descriptors of the steric, electrostatic, H-bond donor and H-bond acceptor field descriptors is considered as the optimal one, which has a *Q*^2^ value of 0.587, a high *R*^2^_ncv_ value of 0.900, a high *F* value of 94.879 and a low SEE value of 0.161 with 7 optimum components, implying a good internal predictability. And the relative contributions of the steric, electrostatic, H-bond donor and H-bond acceptor fields are 24.5%, 45.4%, 14.9% and 15.2% in turn. The higher contribution of the electrostatic field indicates that electrostatic feature plays more roles in the antergic activity for the series.

**Figure 3:**
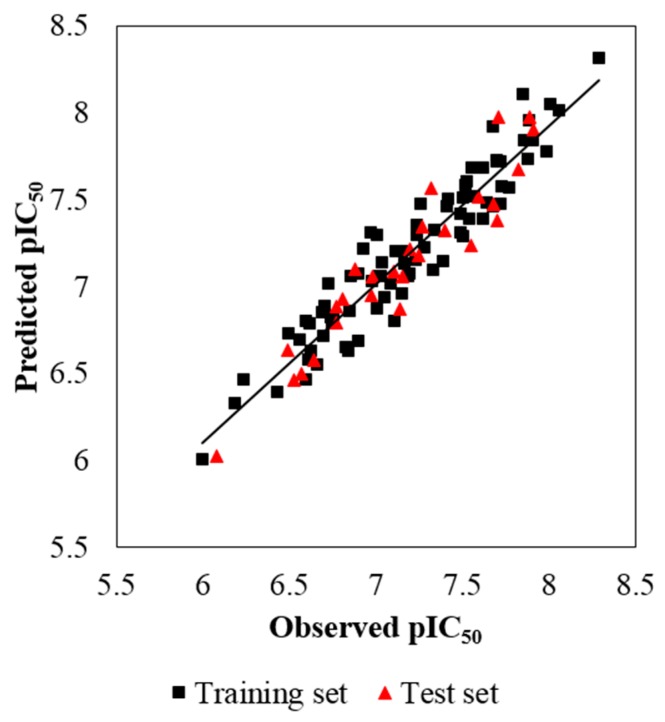
The scatter plot of the predicted versus the experimental pIC_50_ values using the training (filled black squares) and the test (filled red triangles) compounds from the final optimal CoMSIA model. The solid line is the regression line for the predicted and experimental activities of training and test sets.

**Figure 4:**
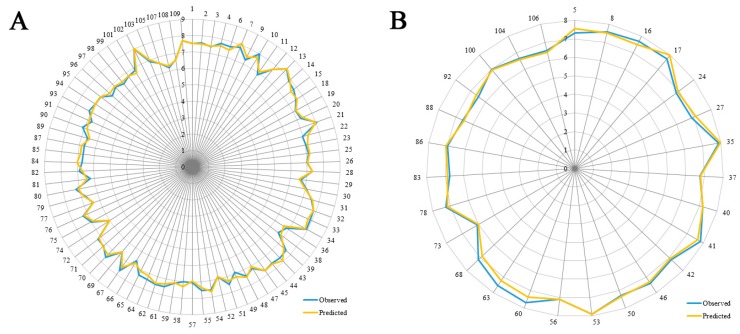
The radar plots of the predicted versus the experimental pIC_50_ values using the training (**A**) and the test (**B**) compounds from the final optimal CoMSIA model.

Some models are found to have admissible internal predictability but unfavorable external predictability [21]. Thus, the *R*^2^_pre_ should also be considered for a reliable model. In this study, a test set of 27 compounds, representing 32.9% of the training set, was employed to validate the robustness of the models. In general, the *R*^2^_pre_ above 0.6 is an acceptable standard [56]. The developed CoMSIA model gives a high *R*^2^_pre_ value of 0.897, much higher than this criterion. Therefore, the CoMSIA model is selected as the optimal one, and its correlation for the whole dataset is described in both scatter plot (Figure 3) and radar plots (Figure 4). As we can see, all the data points distribute uniformly around the regression line in Figure 3, and the data lines overlap within the low deviation in both Figure 4A,B, which illustrate the good correlation of the predicted bioactivity data versus the experimental data, as well as the wonderful predictability and reliability of the obtained CoMSIA model.

### 3.2. CoMSIA Graphical Interpretation

The optimal CoMSIA model developed by steric, electrostatic, H-bond donor and H-bond acceptor field descriptors is further discussed here. And in order to facilitate the analysis, the most active compound **13** of the whole data set is depicted superimposed with the four fields respectively.

**Figure 5:**
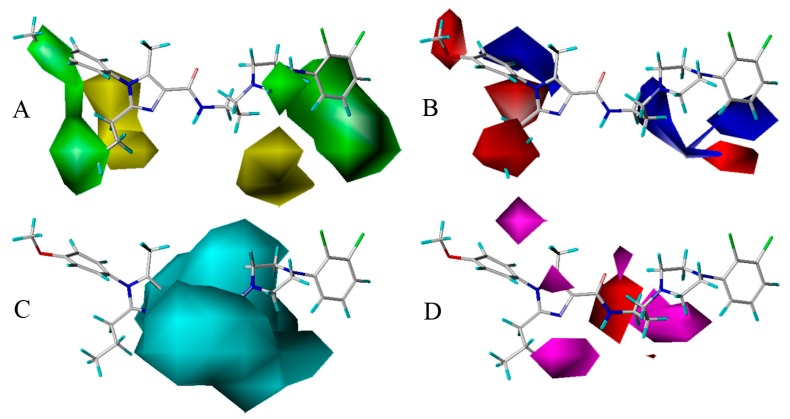
CoMSIA “StDev*Coeff” contour maps. (**A**) Steric (green/yellow) map. Green contours imply regions where bulky groups are beneficial for the activity, and yellow contours imply regions where bulky groups are detrimental for the activity; (**B**) Electrostatic (blue/red) map. Blue contours represent where positive charges promote the affinity, and red contours represent where negative charges promote the affinity; (**C**) H-bond donor (cyan/purple) map. Cyan contours reveal where H-bond donors increase the activity, and purple contours reveal where H-bond donors decrease the activity; (**D**) H-bond accepter (purple/red) map. Purple contours represent regions where H-bond acceptors are beneficial for the activity, while red contours represent regions where H-bond acceptors are detrimental for the activity.

Figure 5A is the steric contour plot, in which the green and yellow isopleths representing 80% and 20% contribution levels each, implying the increased and decreased activity of molecules with the bulky groups, respectively. On one hand, the long green contour around R_1_ substituent position on imidazole ring (ring A) suggests that bulk group is preferred in this position for increasing the antagonistic activity. For example, compounds **21** (pIC_50_ = 8.064), **1** (7.509) and **9** (7.260) have similar structures except that their R_1_ groups are substituted separately by 2,3-dihydrobenzo[*b*][1,4]dioxine, benzene and cyclopentane, displaying the antagonistic activity reducing with the dimensional decrease in R_1_ group. In addition, there is another large green contour around the benzene ring (ring C) showing a steric favored region. Thus, the result occurs for compounds **25** and **24**, in which **25** with methyl substituent at R_5_ position is more potent (pIC_50_ = 7.125) than compound **24** (6.812) with Cl-atom at the same location in activity. On the other hand, a medium sized yellow contour map appears at R_2_ position, revealing the disfavor of bulky group for the antagonistic activity. For instance, compound **48** (pIC_50_ = 7.420) is relatively more active than **50** (7.268) because the substituents of them are methyl and propyl groups, respectively. So is the pair of compounds **40** (pIC_50_ = 7.203) and **42** (7.150). Furthermore, another medium sized yellow contour shows below the piperazine ring (ring B), signifying that the introduction of bulky group to this position would lead to a decrease of the activity.

The electrostatic contour map is displayed in Figure 5B, where blue contours (80% contribution) account for electropositive favorable regions, but red contours (20% contribution) represent electronegative favorable ones. Two blue contours with medium size are observed near R_3_ position and ring B, which display the key role of these sites in electron-donating groups. Take compounds **51** and **52** for example, in which **51** with methyl group at R_3_ has higher activity (pIC_50_ = 7.509) than compound **52** (6.851) with H-atom at the same region. Moreover, there are four medium sized red electronegative regions stumbled on the R_1_, R_2_ positions and ring C, suggesting the favor for electron-withdrawing groups at this location. For example, compound **53** (pIC_50_ = 7.914) with R_1_ substituent of p-F-phenyl is more active than compound **32** (7.857) with substituent of phenyl, while compound **43** (7.678) with substituent of p-methoxyl-phenyl shows lower activity than **32** and **53**. This could be explained that the F-atom has electron-withdrawing feature, while the methoxyl group has electron-donating character.

The H-bond donor contours overlapped on compound **13** are depicted in Figure 5C, where the cyan isopleths (80% contribution) indicate H-bond donors-preferred region, whereas the purple contours (20% contribution) imply H-bond donors-disfavored one. A large cyan contour near the linker chain reveals that H-bond donor substituents will promote the potency. It is observed that the linker position has the NH of amide group, indicating the important roles of the NH playing in donating H to form H-bond interaction with receptors. Actually, this big cyan contour is located around 5-position (N-atom) of ring A and 10-position (O-atom), which signifies the biological activity benefit from decreasing the polarizability of these N-atom and O-atom. Besides, purple contour is hardly found in Figure 5C, implies that the unfavorable H-bond donor interaction may be omitted. This is corresponds well with the experimental results that compounds **68** (pIC_50_ = 7.140) and **90** (7.106) with OH group on the linker chain are more active than unsubstituted molecules **23** (7.041) and **49** (6.928), respectively.

As shown in Figure 5D, the H-bond acceptor contour maps of the CoMSIA model represented by purple (80% contribution) and red (20% contribution) contours depict the H-bond acceptor substituents favored and disfavored regions, respectively. On one hand, three different sized purple contours are observed at ring A, which are consistent with the N-atoms at the 3,5-positions acting as H-bond acceptor. In addition, two other purple contours are observed above the 10-position (O-atom) and ring B, revealing that the introduction of H-bond acceptor groups to the positive influence would lead to a promotion of the inhibitory activity. On the other hand, a red contour appears at the 7-position of linker chain, implying that H-bond donors or no H-bond are preferred at this region. This can be illustrated by the N-atom at the 7-position of linker chain acting as H-bond donor, which is associated with Figure 5C.

### 3.3. Docking Results

**Figure 6:**
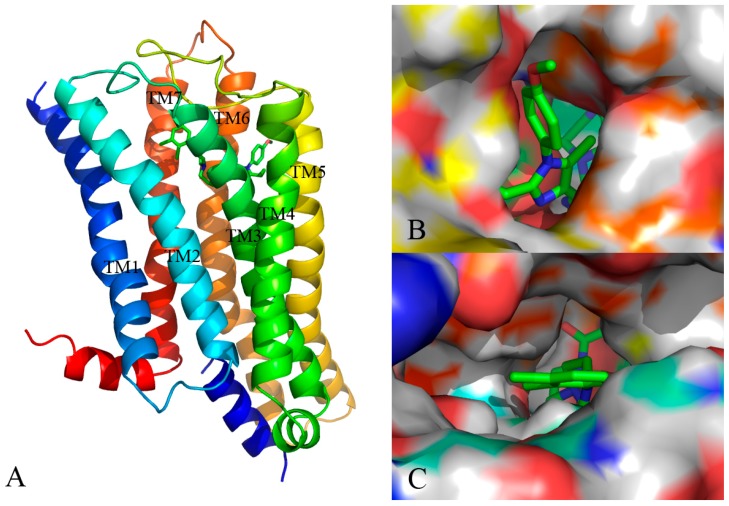
(**A**) The binding pattern of compound **13** and protein; (**B**,**C**) Surface structure depicted with pocket depth potential of the active site within the ligand.

Docking simulation is often used in drug design to predict the optimal orientation of a ligand as well as to elucidate the interactive patterns between the ligand and its target protein [26]. In current work, a computational docking study was conducted and the most optimal conformation was determined by the GoldScore value. As seen from Figure 6, the binding pattern of the most efficacious compound **13** into the active site of the protein is shown.

As displayed in Figure 6A, the 5-HT_2A_ receptor sequence is roughly composed of seven transmembrane helices (TM1–TM7), in which the binding site of the ligand-receptor complex is observed to be located among five transmembrane helices (TM2–TM3, TM5–TM7) and embedded within the upper half of the helical domain. As shown in Figure 6B,C, the ring A of compound **13** is located at the lateral entrance of this binding cavity, and the linker chain, rings B and C are tightly confined in the narrow top of the pocket taken shape by the receptor interface. Indeed, the scaffold of compound **13** is bent at the linker position, and strong interaction is formed between surrounding residues and the distorted ring B, implying the resistance encountered by ring B. Furthermore, ring C takes up sufficient room with near residues. Actually, in our previous contour distribution map (Figure 5A), the presence of a middle-sized yellow contour below ring B and a large green contour around ring C is in accordance with this docking result. Moreover, at the inlet location the *p*-methoxyl-phenyl is flexible to extend deeper into the pocket while the n-propyl taking up most of the narrow space around seems relatively steady, revealing that introduction of a bulky substituent around R_1_ and near R_2_ favors and disfavors the biological activity, respectively, which is also in agreement with the green and yellow contours along ring A as shown in Figure 5A.

**Figure 7:**
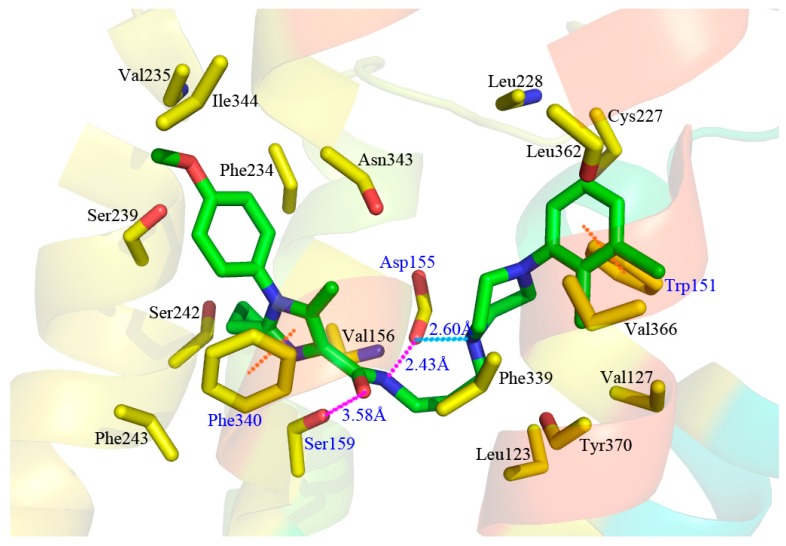
Compound **13** and the interaction groups of the key amino acids are shown in sticks and highlighted with green and yellow carbons, respectively. N-atoms, O-atoms and S-atoms are colored blue, red and yellow, respectively. H-bond, π-π stacking and electronic interactions are shown as purple, orange and azure dashed lines, respectively.

A detailed inspection of the docked complex of 5-HT_2A_ receptor reveals the ligand’s binding conformation and corresponding interaction mechanism as demonstrated in Figure 7, which depicts the constitution of the binding pocket, including all those significant residues as Trp151, Asp155, Ser159 and Phe340. Clearly, four factors, namely hydrophobic, H-bond, electronic and π-π stacking interactions, are observed contributing to the intimate interaction of the antagonist with the target receptor. As a matter of fact, among the 20 amino acid residues (within 4.5 Å distance from the ligand) of the interface, hydrophobic residues account for about two-thirds proportion, which help induce the active conformation of antagonists. For example, ring C occupies a hydrophobic pocket consisting of Val127, Trp151, Cys227, Leu228, Leu362 and Val366, so the hydrophobic interaction plays an important role there. Meanwhile, the three hydrophobic phenylalanine residues (Phe234, Phe243 and Phe340) near R_1_ group of ring A imply bulky groups favorable of this region, which is in accordance with the results of Figure 5A. Furthermore, at the corner of the pocket, both hydrophobic and hydrophilic resides (Leu123, Asp155, Val156, Ser159 and Phe339) locate around the linker chain. In this area, the O-atom of the amide forms one H-bond with the side chain of Ser159 (–O···HO–, 3.58 Å), and the NH forms another H-bond with the side chain of Asp155 (–NH···O–, 2.43 Å). This is well consistent with the presence of the large cyan and the small purple contours above the linker chain in Figure 5C,D. At the same time, the electropositive N-atom of ring B interacts with the electronegative O-atom of Asp155 (–N···O–, 2.60 Å), which corresponds well with the medium-sized blue contour near the ring B as shown in Figure 5B. Besides, the residues Trp151 and Phe340 respectively form edge-to-face π-π stacking and face-to-face π-π stacking interactions with ring C and ring A, which are also helpful in anchoring antagonists in the cleft.

To look at Figure 7 as a whole, compound **13** inserts into the protein chain like an expansive “V”, which is composed of two (right and left) branches and one vertex corner. The right branch of the “V” firstly extends to a hydrophobic cage and is further anchored by a π-π stacking with Trp151. Then, the corner of “V” made of linker chain is fixed by H-bonds with Asp155 and Ser159, and is bent to fit for the electronic interaction with residue Asp155. Finally, ring A, together with its three substitutional groups constitutes the left branch, which stretches to the cavity and is grabbed by the π-π stacking with residue Phe340. All in all, the π-π stacking, H-bond, electronic and hydrophobic interactions mentioned above altogether form the active conformation of compound **13**. Furthermore, the results generated by both docking and 3D contour maps are complementary and validated each other, revealing that the QSAR model is rational and could provide a lot of useful information for designing novel long chain arylpiperazine derivatives as 5-HT_2A_ antagonists.

### 3.4. Molecular Dynamics Studies

**Figure 8:**
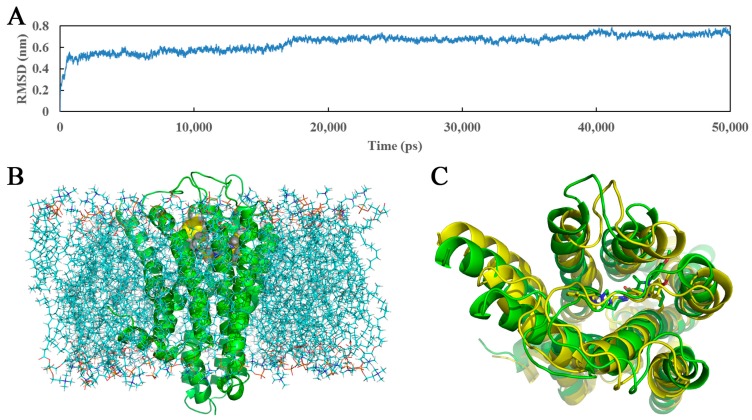
(**A**) The time evolutions of RMSD of the backbone atoms from the initial docked structure during the 50,000 ps MD simulations; (**B**) The ligand-receptor complex embedded into the lipid bilayer after 50,000 ps of MD simulation. Ligand, receptor and lipid molecules are shown as yellow spheres, green cartoon and blue lines, respectively; (**C**) The projection of the superimposed backbone atoms of the average structure of the last 10,000 ps of the MD simulation (yellow) and the initial structure (green). Compound **13** is respectively represented as carbon-chain in green for the initial complex and yellow for the average structure.

Unlike molecular docking, which regards proteins as relatively rigid structures and neglects their conformational flexibility, MD simulations computationally probe the dynamics and structure of biological macromolecules and seem more reliable with a view to the protein flexibility, providing the atomic-level changes of the docked complex structure [57,58]. In this work, the docked complex of 5-HT_2A_ receptor as starting molecular structure was further used to undertake a 50,000 ps MD simulation in a DOPC lipid bilayer to explore the dynamical image of the conformational diversity of the ligand binding to the receptor. The snapshot of the system after 50,000 ps MD simulations is shown (Figure 8B). For ensuring the rationality of the sampling method and exploring the dynamic stability of the ligand-protein system, root-mean-square deviation (RMSD) was calculated as a geometric measure of the conformational alterations from the initial structure, ranging from 1.1 to 8.0 Å in Figure 8A. It is clearly observed that the RMSD of the system retains around 7.2 Å after the initial 17,000 ps of free equilibration and reaches this converged stage throughout the following simulation, indicating the equilibrated conformation of the docked complex structure. Generally, compared with the use of a single crystal structure, the adoption of the MD average structure is considered more helpful and reliable [59]. Thus, presently Figure 8C is drawn to display the average structure (yellow) of the last 10,000 ps in the MD simulation superposed by the initial docked structure (green), and the initial and the final average structures of compound **13** are depicted in green and yellow sticks, respectively.

**Figure 9:**
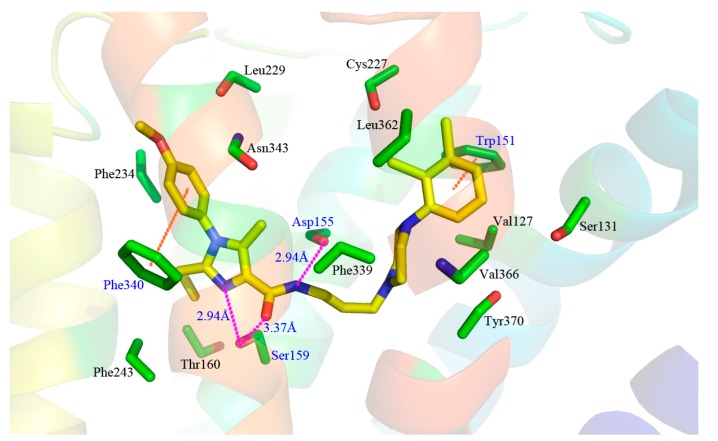
Compound **13** and the interaction groups of the key amino acids are depicted in sticks and highlighted with yellow and green carbons, respectively. N-atoms, O-atoms and S-atoms are colored blue, red and yellow, respectively. H-bond and π-π stacking interactions are represented as purple and orange dashed lines, respectively.

As noticed in Figure 8C, the average structure derived from MD simulation is in well agreement with the docked model of the complex, which validates the rationality of the docking model. Whereas, a slight conformational deviation that the ring A of MD average structure extends a little more expansively than that of the docking ligand, is also observed. In view of the possible interaction variation between compound **13** and 5-HT_2A_ receptor derived from the variety and mobility of the complex system when compared with the docking results, the binding mode extracted from the MD simulation was also investigated in terms of π-π stacking, H-bond and hydrophobic interactions as shown in Figure 9.

Obviously, the majority of crucial amino acid residues from the average complex structure obtained in MD simulation are greatly similar to those of the docking analysis. In accordance with the above docking results, the right branch of compound **13** is also anchored in a hydrophobic cleft constituted by a multitude of hydrophobic residues, such as Val127, Trp151, Leu362, Val366 and Tyr370. Moreover, the essential edge-to-face π-π stacking between Trp151 and ring C is predictably retained in MD results. In addition, one H-bond between the NH of the amide of compound **13** and Asp155 (–NH···O–, 2.94 Å) and another H-bond between the O-atom and Ser159 (–O···HO–, 3.37 Å) on vertex corner of the “V” are also formed in the system which tend to play necessary roles in the stabilization of the antagonists within the active site of the target protein. Although the above reproductions of binding interactions as revealed in the MD analysis further support the docking discovery, it is worth mentioning that slight discrepancy arises in the putative cavity. Due to the approximately more expansive conformation of compound **13** during MD simulation, the difference in electronic and face-to-face π-π stacking formations is observed. The electronic interaction between the electropositive N-atom of ring B and the electronegative O-atom of Asp155 in previous docking results is broken, so is the face-to-face π-π stacking between Phe340 and ring A. Meanwhile, two new interactions, including the H-bond between the N-atom at 5-position of ring A and Ser159 (–N···HO–, 2.94 Å), and the edge-to-face π-π stacking between Phe340 and the phenyl of R_1_, anchor the left branch of the “V” in the cleft. As a matter of fact, the variation of these two stretching formations leads to a much wider-open conformation of compound **13** in MD results. In a word, despite the subtle changes, the ligand is relatively stable at the active site of the receptor, and the MD analysis agrees well with the docking model, implying the rationality of the docking analysis in terms of reliability and helpfulness for optimization and design of potent 5-HT_2A_ antagonists.

### 3.5. Docking Comparison

**Figure 10:**
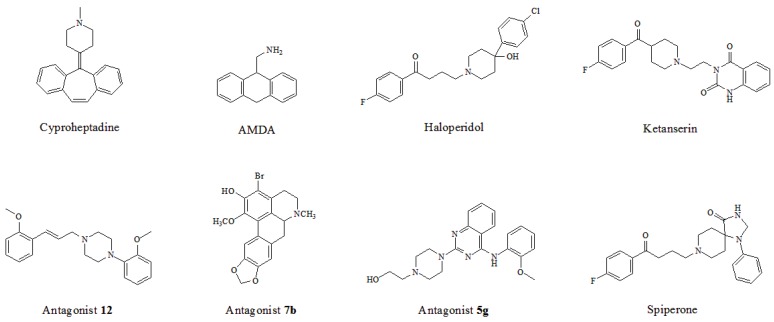
Eight known 5-HT_2A_ antagonists binding in the 5-HT_2A_ receptor docking model.

By a combinatorial study using both the docking and MD analyses, as well as a detailed comparison between these results, it is demonstrated that the present docking model is reliable in reflecting the particular binding mechanism in the 5-HT_2A_ receptor active site. To further explore the interaction features of general 5-HT_2A_ antagonists and the binding model of arylpiperazine derivatives with the target protein, a comparison of previously-reported docking studies of known 5-HT_2A_ antagonists with the ones assessed in our current work was conducted [39,40,41,42,43,44,45,46]. Above Table 2 summarizes the important information obtained from these studies, for purpose to compare their similarities and differences, with their representative structures of the ligands summarized in Figure 10.

**Figure 11:**
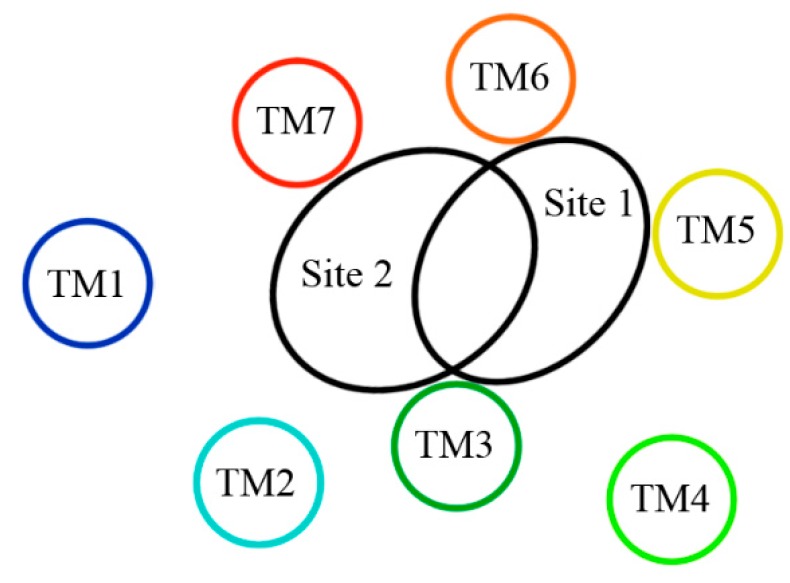
The sterically accessible binding pockets within the 5-HT_2A_ receptor regarded as Site 1 (agonist site) and Site 2 (antagonist site) [40,60].

Up to now, two 5-HT_2A_ receptor binding pockets (listed as Site 1 and Site 2, respectively) have been proposed [40,60,61]. As noted in these literatures, Site 1 (TM3 flanked by TM4, TM5, and TM6) has been identified as an agonist binding site, and Site 2 (TM3 flanked by TM2, TM6, and TM7) has been identified as an antagonist binding site [40,60,61]. As illustrated in Figure 11, there is some overlap between Site 1 and Site 2, and the common region between these sites involves residues that are both parts of helices TM3 (Asp155 and Ser159) and TM6 (Trp336 and Phe339). [40]. In terms of difference of these sites, it mainly lies in those residues which participate in the interactions of the ligand-receptor, such as H-bond, π-π stacking and hydrophobic interaction, etc. Analysis of Table 2 leads us to consider a conventional antagonist binding cavity (Site 2), where these binding modes share same characteristics with those H-bond interactions and simultaneously most of the ligands occupy approximately similar areas constituted by hydrophobic residues.

Observably, in the case of the amino acid profile settled around compound **13**, the pocket that the small ligand is docked into conforms well to Site 2. Besides, with regard to all docking structures of these antagonists that bind to Site 2, their docking modes can be divided into two types, namely plane or long-chained configurations.

**Figure 12:**
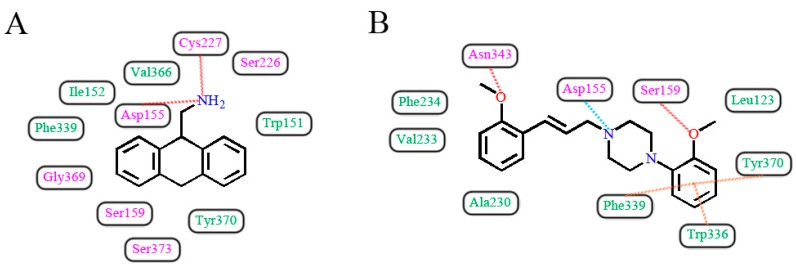
Two docking modes in the 5-HT_2A_ receptor. (**A**) AMDA [40]; (**B**) Antagonist **12** [43]. H-bond, π-π stacking and salt bridge interactions are observed as red, orange and blue dashed lines, respectively. Hydrophobic and hydrophilic residues are respectively displayed as green and purple words.

In light of those compounds adopting the fist docking mode, i.e., the plane configuration, they mainly include dibenzocycloheptatriene, dihydroanthracene and aporphine derivatives, represented by cyproheptadine, AMDA and antagonist **7b**, respectively. For instance, AMDA is an organic compound as a selective antagonist for 5-HT_2A_ receptor, and the binding mode of AMDA has been disclosed by Runyon et al. (No. 2) in Figure 12A, where the homology model was built based on the crystal structure of bovine rhodopsin (PDB entry 1U19:A) used as a template [40]. As shown in Figure 12A, the proposed binding mode of AMDA encompasses H-bonding and hydrophobic interactions. The ammonium substituent of AMDA interacts with not only Asp155 but also the backbone carbonyl O-atom of Cys227. Furthermore, the docked ligand solution also forms close hydrophobic contacts with Val366 [40]. In terms of other plane-type 5-HT_2A_ antagonists, cyproheptadine from the work of Westkaemper et al. (No. 1) and antagonist **7b** from the work of Ponnala et al. (No. 6) are both located at Site 2 which contains hydrophobic residues Phe339 and Phe340, and a strong H-bond is also formed between the prominent residue Asp155 and ligand.

As to those molecules adopting the second binding mode, the long-chained configuration, they mostly contain piperidine and piperazine derivatives, such as haloperidol, ketanserin, antagonist **12**, antagonist **5g** and spiperone. For example, Sencanski et al. (No. 5) utilized a docking process with a homology model of 5-HT_2A_ receptor constructed with the crystal structure of β_2_-adrenergic receptor (PDB entry 3D4S) [43]. Antagonist **12**, a long-chained arylpiperazine-like ligand, was investigated on its binding mode in their work, as depicted in Figure 12B. Their docking results show that antagonist **12** forms various interactions with 5-HT_2A_ receptor: H-bond with Asn343, salt bridge between ligand and Asp155, and another H-bond with Ser159 and multiple π-π interactions with the binding pocket formed by Trp336, Phe339 and Tyr370 (Figure 12B), which has some similarities with our docking result. Moreover, hydrophobic residues surrounding the other parts of antagonist **12**’s structure include Leu123, Ala230, Val233 and Phe234. Therefore, due to the fact that antagonist **12** and compound **13** belong to the same class of arylpiperazines, their binding constitutions in 5-HT_2A_ receptor are similar. In addition, by docking summary of other long-chained ligands, a few similar effects like H-bond, π-π stacking, salt bridge, cation-π and hydrophobic interaction are also observed in No. 3, 4, 7, 8, 9 as listed in Table 2. All these interactions tend to be necessary for the high-affinity binding of piperidin and piperazine derivatives.

In summary, currently (1) all 5-HT_2A_ antagonists are observed binding to Site 2 instead of Site 1 of the receptor regardless of their various structural scaffolds, and (2) they adopt one of the two binding modes, i.e., plane or long-chained configurations when interacting with the target; meanwhile (3) the arylpiperazine derivatives studied presently also bind to Site 2 as 5-HT_2A_ antagonists, in an approximately “V” conformation stabilized by the complex interactions including H-bond, π-π stacking, hydrophobic, and even electronic interplays which also belongs to the long-chained one in configuration. All these findings could provide insights for the future exploitation of novel 5-HT_2A_ antagonists with high inhibitory activities.

### 3.6. Design of New 5-HT_2A_ Antagonists

**Figure 13:**
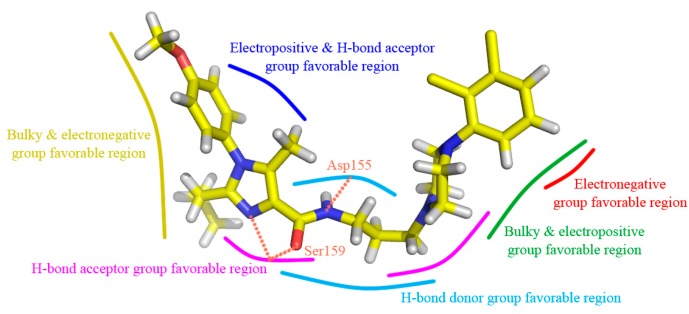
Crucial interaction features of template compound **13** with 5-HT_2A_ receptor.

**Figure 14:**
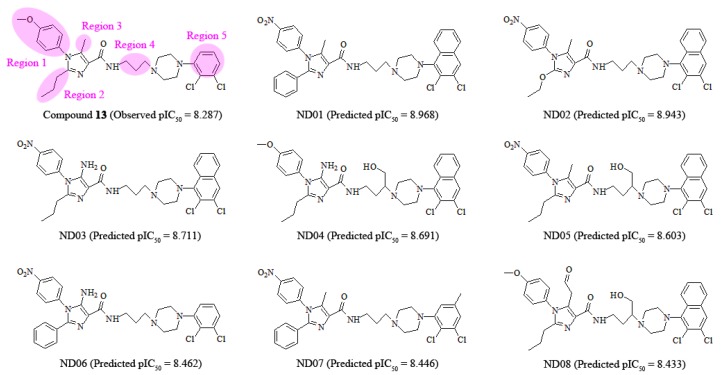
Structures and predicted activities of newly designed 5-HT_2A_ antagonists.

According to 3D-QSAR, docking and MD results, the arylpiperazine derivatives’ crucial structural actors influencing the antagonism potency are intuitively summarized in Figure 13. Based on this information, we further designed a set of new molecules and estimated their potential activities using our optimal CoMSIA model. At present, these modifications are concentrated on regions 1–5 in the most potent compound **13** as the template. As revealed in Figure 14, eight molecules (ND01-08) possess potent antergic activities against 5-HT_2A_ receptor and larger pIC_50_ than that of the template compound **13**.

Since our present work mainly focuses on the in silico study of the SAR of arylpiperazine derivatives as 5-HT_2A_ antagonists and the exploration of their binding mechanism by modeling results, the experimental evaluation for verifying these novel-designed molecules’ activities is our further work.

## 4. Conclusions

In the current work, to investigate the specific binding modes of 109 arylpiperazine scaffold-based 5-HT_2A_ antagonists with the target protein, a comprehensive in silico study was carried out by an integrated use of a series of computational techniques including 3D-QSAR analysis, molecular docking and MD simulation. Our findings are summarized as follows:

(1) The optimal CoMSIA model exhibits a statistically predictable ability with *Q*^2^ = 0.587, *R*^2^_ncv_ = 0.900 and *R*^2^_pre_ = 0.897, proving its wonderful reliability and predictability.
(2) Bulky groups at R_1_ position and ring C, electropositive substituents at R_3_ and ring B, electronegative groups at R_1_, R_2_ positions and ring C, H-bond donor substituents at linker chain, H-bond acceptor groups at 10-position, ring A and ring B are favorable to increase the inhibitory activity.
(3) The scaffold of antagonists fits into the conventional Site 2 of 5-HT_2A_ receptor with an approximately “V” conformation and follows the second binding mode, which is fixed by three H-bonds with Asp155 and Ser159, two π-π stacking interactions with Try151 and Phe340, and hydrophobic interaction.
(4) Several new potential arylpiperazine antagonists of 5-HT_2A_ receptor were also newly-designed based on these results.

All in all, the above CoMSIA model, docking and MD obtained results associate well with each other, and all these in silico models may facilitate the modification and design of novel 5-HT_2A_ antagonists as promising antidepressant drugs.

## Figures and Tables

**Table 1:** Representative molecular structures and IC_50_ values of arylpiperazine derivatives.

No.	Structure	IC_50_ (nM)	No.	Structure	IC_50_ (nM)
**2**	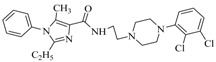	27	**5 *^t^***	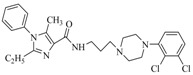	48
**13**	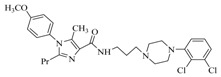	5.17	**18**	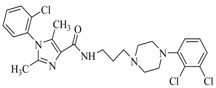	30
**32**	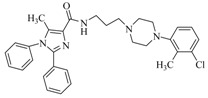	13.9	**46 *^t^***	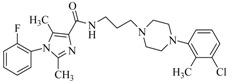	40
**47**	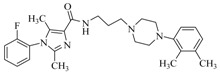	64.8	**49**	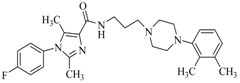	117.9
**60** *^t^*	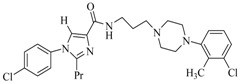	20	**74**	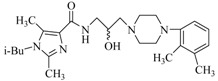	1011
**84**	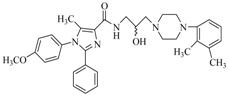	185	**90**	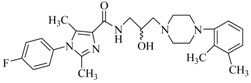	78.3
**95**	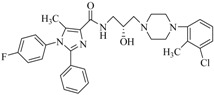	95	**101**	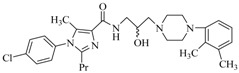	147
**105**	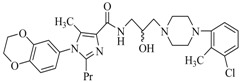	237	**106 *^t^***	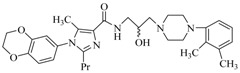	294

*^t^* Compounds belonging to the test set.

**Table 2:** The summary of various ligand-5-HT_2A_ receptor docking studies.

No.	Researchers	Template	Representative Structure	Site Type	Binding Interactions	Crucial Residues
1	Westkaemper et al. [39]	GF-62 cells	Cyproheptadine	Site 2	H-bond	Asp155
2	Runyon et al. [40]	Bovine rhodopsin (PDB entry 1U19:A)	AMDA	Site 2	H-bond, hydrophobic interaction	Asp155, Cys227, Val366
3	Kanagarajadurai et al. [41]	β_2_-adrenergic receptor (PDB entry 2RH1)	Haloperidol	Site 2	H-bond, hydrophobic interaction	Asp155, Phe339, Phe340, Tyr370
4	Yap et al. [42]	β_2_-adrenergic receptor (PDB entry 2RH1)	Ketanserin	Site 2	H-bond	Thr134, Asp155, Phe234, Val235, Ser239
5	Sencanski et al. [43]	β_2_-adrenergic receptor (PDB entry 3D4S)	Antagonist **12**	Site 2	H-bond, π-π stacking, salt bridge	Asp155, Ser159, Trp336, Phe339, Asn343, Tyr370
6	Ponnala et al. [44]	β_2_-adrenergic receptor (PDB entry 2RH1)	Antagonist **7b**	Site 2	H-bond, hydrophobic interaction	Asp155, Phe339, Phe340
7	Deng et al. [45]	β_2_-adrenergic receptor	Antagonist **5g**	Site 2	H-bond, cation-π	Trp151, Asp155, Ser159, Tyr370
8	Gandhimathi et al. [46]	β_2_-adrenergic receptor (PDB entry 2RH1)	Spiperone	Site 2	H-bond, salt bridge	Asp155, Asn363
9	Gandhimathi et al. [46]	β_2_-adrenergic receptor (PDB entry 3SN6)	Spiperone	Site 2	H-bond, π-π stacking	Trp151, Asp155, Asn343

**Table 3:** Summary of 3D-QSAR results.

**PLS Statistics**	**CoMFA**	**CoMSIA**
*Q*^2^	0.496	0.587
*R*^2^_ncv_	0.914	0.900
*R*^2^_pre_	0.831	0.897
*F*	132.803	94.879
SEE	0.148	0.161
SEP	0.402	0.339
ONC	6	7
	**Field Contribution/%**	
Steric	55.6	24.5
Electrostatic	44.4	45.4
H-bond donor	-	14.9
H-bond acceptor	-	15.2

*Q*^2^: cross-validated correlation coefficient; *R*^2^_ncv_: non-cross-validated correlation coefficient; *R*^2^_pre_: predicted correlation coefficient; *F*: ratio of *R*^2^_ncv_ explained to unexplained, *F* = *R*^2^_ncv_/(1 − *R*^2^_ncv_); SEE: standard error of estimate; SEP: standard error of prediction; ONC: optimal number of components.

## Supplementary Materials

The following are available online. Table S1: Molecular structures and pIC_50_ values of arylpiperazine derivatives.
