# Supplementary material for: Mechanism Exploration of Arylpiperazine Derivatives Targeting the 5-HT2A Receptor by In Silico Methods
Source: Molecules. 2017 Jun 26;22(7):1064. doi: 10.3390/molecules22071064 (PMC6152085; doi:10.3390/molecules22071064)
Supplement: Supplementary file 1 [file molecules-22-01064-s001.pdf]

# Mechanism Exploration of Arylpiperazine Derivatives Targeting the 5-HT$_{2A}$ Receptor by In Silico Methods

**Feng Lin [1,2], Feng Li [3], Chao Wang [2], Jinghui Wang [2], Yinfeng Yang [2], Ling Yang [4] and Yan Li [1,2,*]**

1 Key Laboratory of Xinjiang Endemic Phytomedicine Resources, Pharmacy School, Shihezi University, Shihezi 832002, Xinjiang, China; fenglin_dut@yeah.net
2 Key Laboratory of Industrial Ecology and Environmental Engineering (MOE), Faculty of Chemical, Environmental and Biological Science and Technology, Dalian University of Technology, Dalian 116024, Liaoning, China; chaowang_dlou@163.com (C.W.); jhwang_dlut@163.com (J.W.); yinfengyang@yeah.net (Y.Y.)
3 Department of Civil Engineering, Henan Institute of Engineering, Zhengzhou 451191, Henan, China; fengli@haue.edu.cn
4 Institute of Interdisciplinary Integrative Medicine Research, Shanghai University of Traditional Chinese Medicine, Shanghai 201203, China; yling@dicp.ac.cn
* Correspondence: yanli@dlut.edu.cn; Tel.: +86-0411-8498-6062

**Table S1.** Molecular structures and pIC$_{50}$ values of arylpiperazine derivatives.

| No. | Structure | pIC$_{50}$ (M) | No. | Structure | pIC$_{50}$ (M) |
|---|---|---|---|---|---|
| 1 | 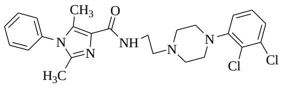 | 7.509 | 2 | 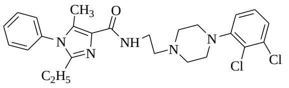 | 7.569 |
| 3 | 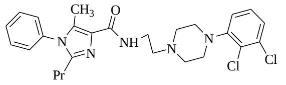 | 7.409 | 4 | 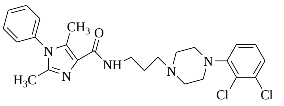 | 7.730 |
| 5[t] | 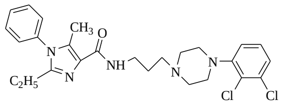 | 7.319 | 6 | 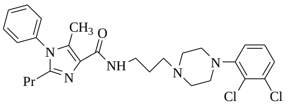 | 7.678 |
| 7 | 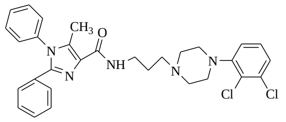 | 7.848 | 8[t] | 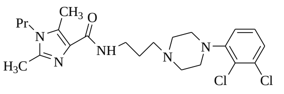 | 7.585 |
| 9 | 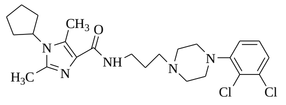 | 7.260 | 10 | 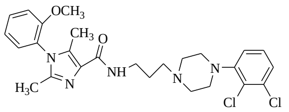 | 7.987 |
| 11 | 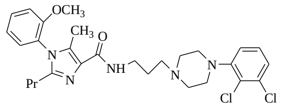 | 6.857 | 12 | 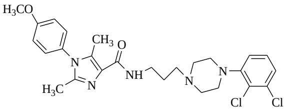 | 7.699 |
| 13 | 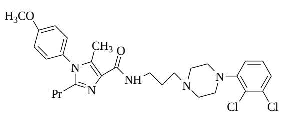 | 8.287 | 14 | 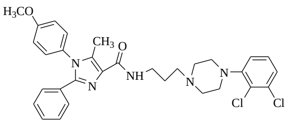 | 7.886 |
| 15 | 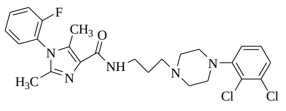 | 7.775 | 16[t] | 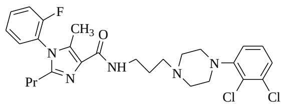 | 7.678 |

| | | | | | |
|---|---|---|---|---|---|
| 17[t] | 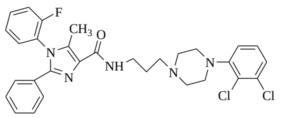 | 7.710 | 18 | 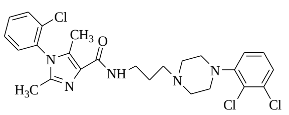 | 7.523 |
| 19 | 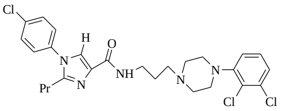 | 7.149 | 20 | 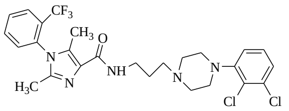 | 7.237 |
| 21 | 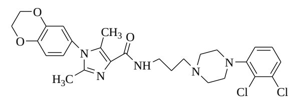 | 8.064 | 22 | 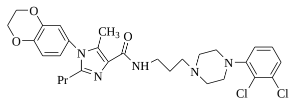 | 6.975 |
| 23 | 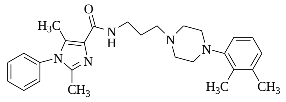 | 7.041 | 24[t] | 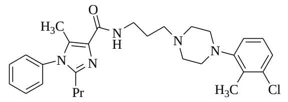 | 6.812 |
| 25 | 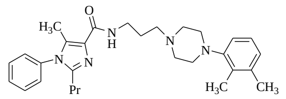 | 7.125 | 26 | 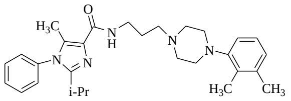 | 6.979 |
| 27[t] | 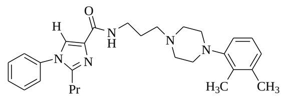 | 6.876 | 28 | 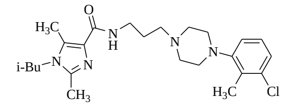 | 7.342 |
| 29 | 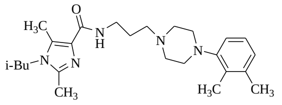 | 6.664 | 30 | 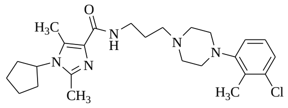 | 7.094 |
| 31 | 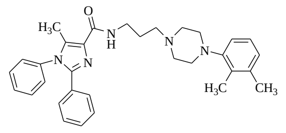 | 7.532 | 32 | 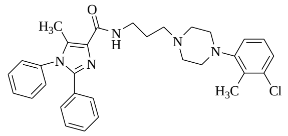 | 7.857 |
| 33 | 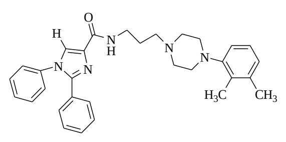 | 7.879 | 34 | 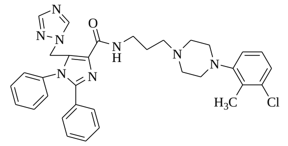 | 7.910 |
| 35[t] | 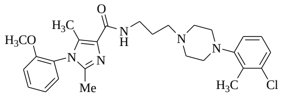 | 7.893 | 36 | 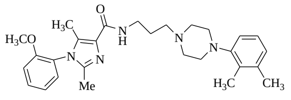 | 6.842 |
| 37[t] | 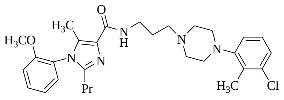 | 6.767 | 38 | 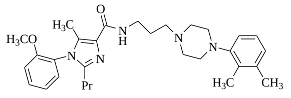 | 6.597 |
| 39 | 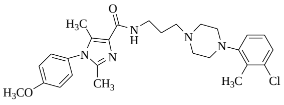 | 7.636 | 40[t] | 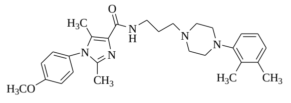 | 7.203 |
| 41[t] | 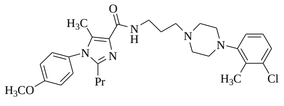 | 7.818 | 42[t] | 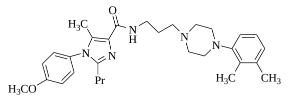 | 7.150 |
| 43 | 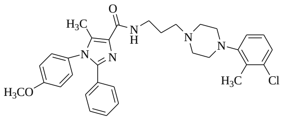 | 7.678 | 44 | 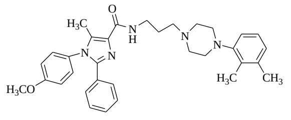 | 7.620 |

| | | | | | |
|---|---|---|---|---|---|
| 45 | 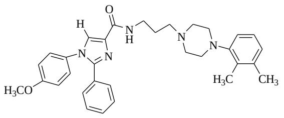 | 7.721 | 46[t] | 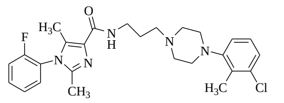 | 7.398 |
| 47 | 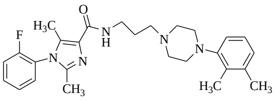 | 7.188 | 48 | 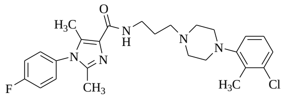 | 7.420 |
| 49 | 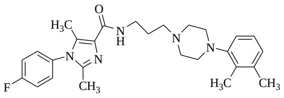 | 6.928 | 50[t] | 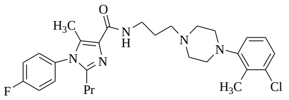 | 7.268 |
| 51 | 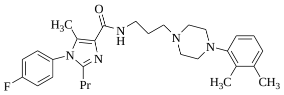 | 7.509 | 52 | 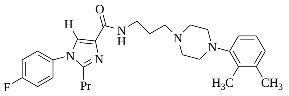 | 6.851 |
| 53[t] | 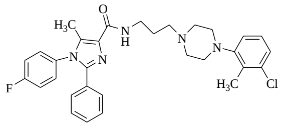 | 7.914 | 54 | 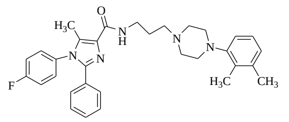 | 7.556 |
| 55 | 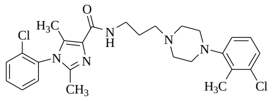 | 7.538 | 56[t] | 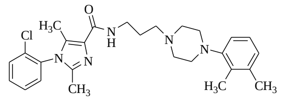 | 7.097 |
| 57 | 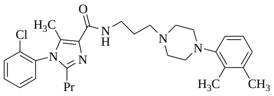 | 7.046 | 58 | 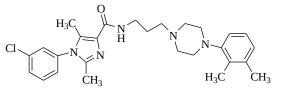 | 7.014 |
| 59 | 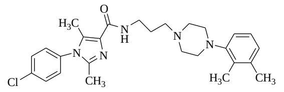 | 7.174 | 60[t] | 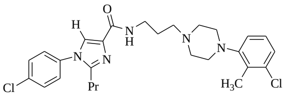 | 7.699 |
| 61 | 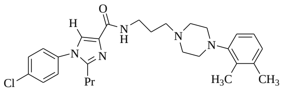 | 7.489 | 62 | 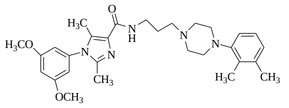 | 7.495 |
| 63[t] | 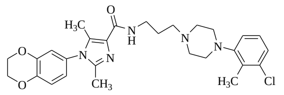 | 7.553 | 64 | 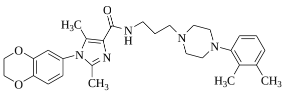 | 7.391 |
| 65 | 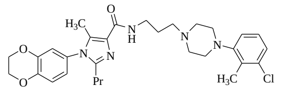 | 7.328 | 66 | 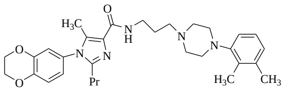 | 6.618 |
| 67 | 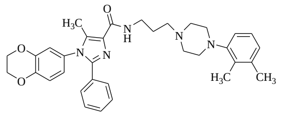 | 7.721 | 68[t] | 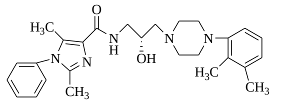 | 7.140 |
| 69 | 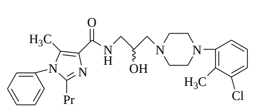 | 6.697 | 70 | 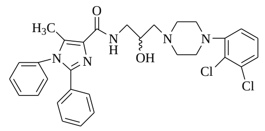 | 7.620 |
| 71 | 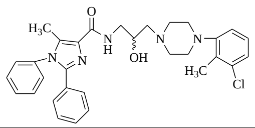 | 7.244 | 72 | 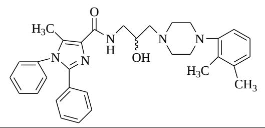 | 7.232 |

| | | | | | |
|---|---|---|---|---|---|
| 73[t] | 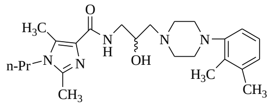 | 6.076 | 74 | 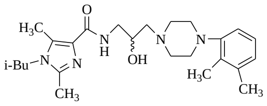 | 5.995 |
| 75 | 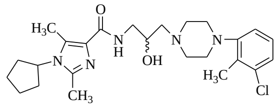 | 7.011 | 76 | 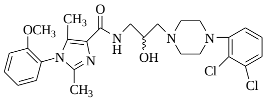 | 7.283 |
| 77 | 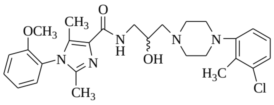 | 6.432 | 78[t] | 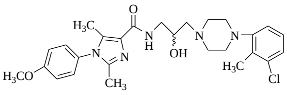 | 7.252 |
| 79 | 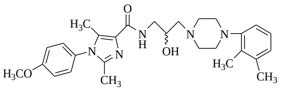 | 6.757 | 80 | 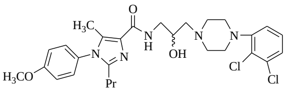 | 7.200 |
| 81 | 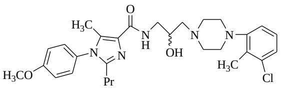 | 6.235 | 82 | 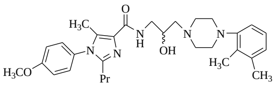 | 6.900 |
| 83[t] | 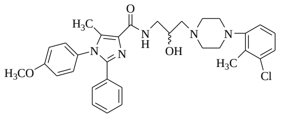 | 6.770 | 84 | 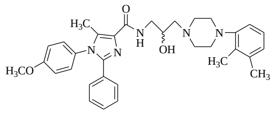 | 6.733 |
| 85 | 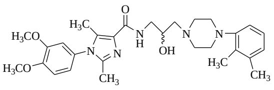 | 6.689 | 86[t] | 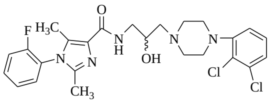 | 6.983 |
| 87 | 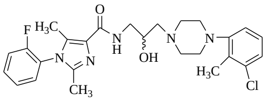 | 6.706 | 88[t] | 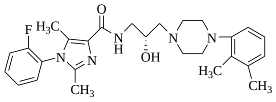 | 6.569 |
| 89 | 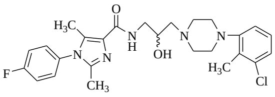 | 6.595 | 90 | 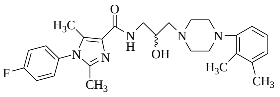 | 7.106 |
| 91 | 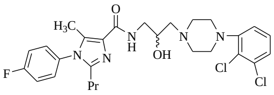 | 6.708 | 92[t] | 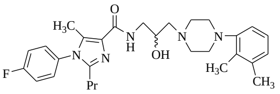 | 6.485 |
| 93 | 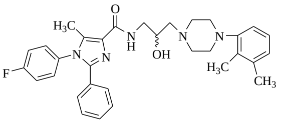 | 7.149 | 94 | 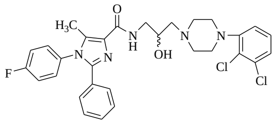 | 7.027 |
| 95 | 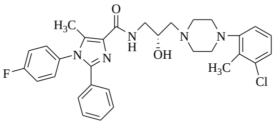 | 7.022 | 96 | 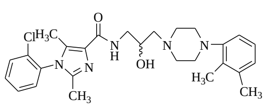 | 6.498 |
| 97 | 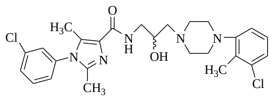 | 6.740 | 98 | 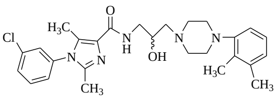 | 6.561 |
| 99 | 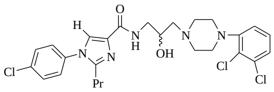 | 6.753 | 100[t] | 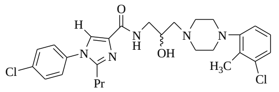 | 6.971 |

| | | | | | |
|---|---|---|---|---|---|
| 101 | 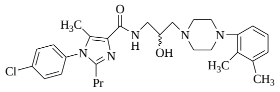 | 6.833 | 102 | 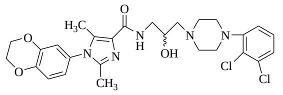 | 8.006 |
| 103 | 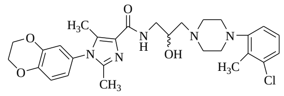 | 6.896 | 104[t] | 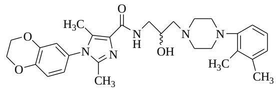 | 6.635 |
| 105 | 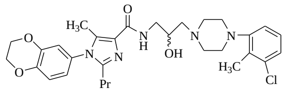 | 6.625 | 106[t] | 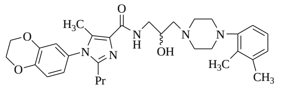 | 6.532 |
| 107 | 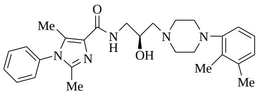 | 6.190 | 108 | 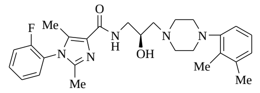 | 6.613 |
| 109 | 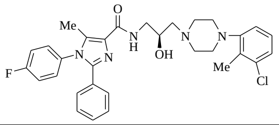 | 7.697 | | | |

[t] Compounds belonging to the test set.
